# Level and Predictors of Knowledge of Reproductive Rights Among Haramaya University Students, Eastern Ethiopia: A Cross-Sectional Study

**DOI:** 10.3389/frph.2021.641008

**Published:** 2021-11-24

**Authors:** Mohammed Yuya, Hassen Abdi Adem, Nega Assefa, Addisu Alemu, Abdurezak Adem Umer, Lemessa Oljira

**Affiliations:** ^1^School of Public Health, College of Health and Medical Sciences, Haramaya University, Harar, Ethiopia; ^2^School of Nursing and Midwifery, College of Health and Medical Sciences, Haramaya University, Harar, Ethiopia; ^3^Department of Public Health, College of Medicine and Health Sciences, Dire Dawa University, Dire Dawa, Ethiopia

**Keywords:** knowledge, reproductive rights, youths, students, Haramaya University, Ethiopia

## Abstract

**Background:** Globally, two-thirds of pre-mature deaths and one-third of the total disease burden in adults are associated with problems that began in adolescent and youth. Global and national acting educational and health policies, strategies, and programs designed to promote, implement, and improve adolescent and youth sexual and reproductive health services utilization should be responsive, consider the knowledge of reproductive rights and risk factors. This study assessed the level and predictors of knowledge of reproductive rights among Haramaya University students in Ethiopia.

**Methods:** An institution-based cross-sectional study was conducted among randomly selected Haramaya University students from March 1 to 24, 2018. A self-administered pre-tested structured questionnaire was used to collect data from participants. Data were entered using EpiData version 3.1 and analyzed using SPSS version 24. Bivariable and multivariable logistic regression analyses were conducted to identify factors associated with knowledge of reproductive rights. Adjusted odds ratio (AOR) with 95% CI was used to report association and significance was declared at *P-*value < 0.05.

**Results:** Of 822 total students invited to the study, 812 (98.8%) respondents participated in the study. A total of 424 students (52.2%, 95% CI: 48.8, 55.4%) had an above-average level of knowledge on reproductive rights. Participants who were in the fourth and above year of the study [AOR = 2.37 (1.58, 3.54)], whose father's had higher education [AOR = 1.89 (1.27, 2.80)], who came from rich families [AOR = 1.54 (1.07, 2.21)], in the health faculty [AOR = 3.37 (2.17, 5.23)], utilized reproductive health services [AOR = 2.81 (2.21, 4.98)] and participated in reproductive health club [AOR = 1.77 (1.27, 2.47)] were significantly associated with knowledge of reproductive rights.

**Conclusion:** Around half of the participants knew reproductive rights. In this study, lack of awareness (information) on reproductive health issues and absence of reproductive health services utilization were strong independent predictors of knowledge of reproductive rights.

## Introduction

Reproductive rights are the basic rights of all couples and individuals to decide freely and responsibly the number, spacing, and timing of their children and to have the information and means to do so, and the right to attain the highest standard of sexual and reproductive health (1, 2). It includes the right to decide on reproduction and sexuality free from discrimination, coercion, and violence (2, 3), and is the cornerstone to empowering people to make informed healthcare decisions (1, 2).

Reproductive right literacy is a key life-saving public health intervention to reduce adolescent and youth morbidity and mortality in today's world (1, 3). Globally, universal access to reproductive right literacy increases opportunities for youth to have informed choice and appropriate decisions regarding their sexual and reproductive well-being, which decreases early marriage and childbirth by 11%, unintended pregnancy by 70%, unsafe abortion by 14%, and HIV/AIDS by 34% (1, 2). In Africa, reproductive right literacy of adolescents and youth prevents the unmet need for contraceptives by 30%, sexual violence by 32%, and HIV/AIDS by 50% (3).

Youths are individuals in a period of transition from childhood to adulthood, between the ages of 15 and 24 years, during which they undergo biological and psychological transitions to attain reproductive capability and become sexually active (4, 5). Inadequate information on reproductive rights exposes youths to multiple problems that have several negative impacts on their health in the adulthood period (6). For instance, unprotected sex, sexually transmitted infections, unwanted pregnancy, violence, and risky behaviors contribute to 17% of the global burden of disease in all ages. Youths are highly vulnerable to unprotected sex, the second leading risk factor for morbidity and deaths in developing countries (2, 3, 7–9). The magnitude of unintended pregnancy is 21% in Ethiopia (10), 26.1% in Addis Ababa, and 41.5% in the Oromia region (11). The median childbearing age range from 15 to 19 years and thus, 45% of total births in Ethiopia occur among adolescent and young women.

In Ethiopian higher education institutions, less than half (46%) of students had reproductive health literacy and 26.9% had knowledge about sexually transmitted infections, 16.5% know about family planning, 14.3% knew access to healthcare services and only 8.6% knew the right to choose when and with whom to have sex (12). In another study, 45.5% of students know reproductive rights; 21.8% had not discussed the sexual and reproductive issues with their partners or healthcare providers or peers (13). A survey conducted in Haramaya University revealed that among sexually active participants, 11.6% had multiple sexual partners and 16.3% of males had sex with commercial sex workers (14). Another study shows, two of six participants did not know the meaning of reproductive health literally (15).

Several barriers that make youth unable to deal with sexual and reproductive violence include shame, guilt, embarrassment, not want to know by friends and family; lack of confidentiality; and fear of being not believed and culturally stigmatized and discriminated (8). The other barrier is lack of information on sexual health among youth may lead to different types of health risks and social problems (16). Globally, studies show higher socioeconomic status is related to the better chance of having sexual and reproductive information (2, 3, 9, 17, 18) through accessing different media and visiting health facilities and seeking related healthcare services, which directly or indirectly invest on sexual and reproductive health issues (2, 3, 8, 9, 17–20). On the other hand, previous studies conducted in Ethiopia were more focused on the utilization of sexual and reproductive health services (18, 20, 21) which could be difficult to increase unless we boosted youth sexual and reproductive health literacy (1–3, 9).

However, overall little information was available on knowledge of reproductive rights and associated factors in Ethiopia and the study setting. In addition, addressing sexual and reproductive health needs and problems of youths has been a priority intervention in population and development policies of low- and middle-income countries including Ethiopia. Moreover, acting worldwide and national educational and public health policies, strategies, and activities developed to promote and improve the level of knowledge of sexual and reproductive health and related rights for the better outcome and fighting preventable adolescents and youth morbidity and deaths would be essential (1–3, 9). Therefore, this study assessed the level and predictors of knowledge of reproductive rights among Haramaya University students in eastern Ethiopia in 2018.

## Materials and Methods

### Study Design and Setting

An institution-based cross-sectional study was conducted at Haramaya University in Ethiopia from March 1 to 24, 2018. Haramaya University is found in the East Hararghe Zone at 510 km East of Addis Ababa, Capital of Ethiopia. Haramaya University, the second oldest University in Ethiopia, was established in 1954. During the study period, University has two campuses, 11 colleges, and 55 departments running 188 academic programs for a total of 16,437 regular students in the undergraduate program's study. University has one teaching tertiary hospital and four medium clinics providing basic health services for the students, staff, and surrounding communities. Haramaya University has different youth clubs such as reproductive health and anti-HIV/AIDs clubs (22).

### Population and Eligibility Criteria

All undergraduate students in Haramaya University were the source population. Randomly selected undergraduate students in Haramaya University during the study period constituted the study population. Students enrolled in the regular undergraduate program and aged 18 years and above were included while critically sick participants who could not respond and absented from the regular class sessions were excluded.

### Sample Size and Sampling Procedure

The sample size was calculated using Epi Info version 7.1 using a single population proportion with the following assumptions: confidence level with 95%, assumed 54.4% proportion of knowledge of reproductive rights among university students (13) 5% margin of error, the significance level of 0.05 design effect of 2 with a 10% non-response proportion. Accordingly, a minimum of 822 students was required to conduct the study.

A multistage stratified sampling technique was used to select the study participants. First, Haramaya University was stratified by campus (Main Campus and Harar Campus) and then, the sample size was proportionally allocated to each campus. Then, allocated samples to each campus was proportionally allocated to their respective college, school, department, and program/unit found under each campus along with their years of the study, using the actual number of learning undergraduate students reviewed from the latest students lists or registrations available in the Associate Registrar Office of Haramaya University during the study period, 2018. We prepared a sampling frame for each faculty/college and finally, the participants were drawn by systematic sampling technique (Supplementary File 1).

### Data Collection Tool and Measurement

Pre-tested structured questionnaires adapted from related published literature (13) were used to collect data from participants. The questionnaire contains information on sociodemographic factors (age, sex, ethnicity, religion, marital status, address, family size, paternal and maternal education, paternal and maternal main occupation, and the wealth index of the family) school-related factors (type of school attended in lower education, college of the study, and year of the study) and reproductive factors (history of sexual experience, age and time at first sex, number of sexual partners, heard about sexual and reproductive health information and their main source of information, participating on the reproductive health club, discussion about sexual and reproductive health issues, and advised on sexual and reproductive health issue and utilization of sexual and reproductive health service) and knowledge of reproductive rights (Supplementary File 2).

#### Knowledge of Reproductive Rights

It was measured using 24 dichotomous yes/no items asking about knowledge of reproductive rights. The response of each item was re-coded “1” when participants responded a right response and “0” point when the participant responded a wrong response and then, composite index score was summed from a total of 24 points, and participants who scored the mean and above were considered to know reproductive rights and not unless otherwise (13).

#### Wealth Index

It was assessed by a standard instrument containing 36 dichotomous (yes/no) items asking about three domains of the family's wealth level (domestic animals, durable assets, other productive assets, and housing conditions). We observed high internal consistency among items (Cronbach α = 0.81) and principal component analysis using the varimax rotation method to compute estimate composite wealth index factors and the wealth status of the participants.

### Data Collection Procedure

Data were collected paper-based through a self-administrated interview conducted over near a month. Ten health professionals holding Bachelor of Science degree in Nursing facilitated the overall data collection and two public health experts holding master's degrees in Public Health were supervised data collection with the principal investigator. The study participants have filled the questionnaire at the same time in lecture halls.

### Data Quality Control

To maintain the data quality, questionnaires were adapted from standard tools and related published works of literature. We pre-tested the tools on 5% of the total sample (41 undergraduate students) to check the validity of the questionnaire in Dire Dawa University, Ethiopia. Data were entered using EpiData version 3.1 software. In field, strict supervision of data collectors and validation of collected data were carried out by supervisors and principal investigators.

### Data Processing and Analysis

After checking for completeness, data were entered into EpiData version 3.1 and analyzed using SPSS version 24. Descriptive statistics such as frequencies, the measure of central tendency, and measures of dispersion were used to characterize the participants. Before any analysis, the internal consistency of items used to measure each composite index was checked by reliability analysis (using Cronbach's α). Principal component analysis with the varimax rotation method was used to estimate composite wealth index score and wealth status of the family of participant. All predictors with *P*-value < 0.25 in the bivariable analysis were considered in our multivariable analysis (23, 24). Multivariable logistic regression analyses were conducted (using the backward stepwise method) to identify factors associated with knowledge of reproductive rights. Adjusted odds ratio (AOR) (95% CI) was used to report strengthen of association and significance was declared at *P*-value < 0.05.

## Results

### Characteristics of Participants

A total of 812 (98.8%) students were involved in the study. About 521 (64.2%) participants were male. The majority, 710 (87.4%) of participants were in the age group of between 20 and 24 years. The mean age ± SD of the participants was 21.35 ± 1.40 years. Four out of 10 (40.6%) participants had mothers who have no formal education, 33.3% were students from a poor family and 72.9% attended their education at governmental schools before joining University (Table 1).

**Table 1:** Sociodemographic characteristics of Haramaya University students, Eastern Ethiopia, 2018 (*n* = 812).

**Characteristic**	**Frequency**	**Percent**
**Age (in years)**		
≤ 19	74	9.1
20–24	710	87.4
≥25	28	3.5
**Sex**		
Male	521	64.2
Female	291	35.8
**Residence area**		
Urban	371	45.7
Rural	441	54.3
**Current marital status**		
Never married	765	94.2
Ever married	47	5.8
**Religion**		
Orthodox	283	34.9
Muslim	272	33.5
Protestant	217	26.7
Others^*a^	40	4.9
**Ethnicity**		
Oromo	398	49.0
Amhara	171	21.1
Sidama	57	7.0
Others^*b^	186	22.9
**Family size**		
≤ 5	180	22.2
>5	632	77.8
**Types of school attended before joining University**		
Private only	110	13.5
Both	110	13.5
Governmental only	592	73.0
**Faculty of the study**		
Institute of technology	297	36.6
Health and medical sciences	133	16.4
Agriculture and environmental sciences	83	10.2
Computing and informatics	68	8.4
Business and economics	62	7.6
Social science and humanities	61	7.5
Natural and computational sciences	46	5.7
Education and behavioral sciences	31	3.8
Law	20	2.5
Veterinary medicine	11	1.3
**Maternal educational level**		
No formal education	349	43.0
Primary education (grade 1–8)	216	26.6
Secondary education (grade 9–12)	131	16.1
Above secondary/higher education	116	14.3
**Paternal educational level**		
No formal education	183	22.5
Primary education (grade 1–8)	249	30.7
Secondary education (grade 9–12)	149	18.3
Above secondary/higher education	231	28.5
**Maternal main occupation**		
Housewife	538	66.3
Employee (government/private)	116	14.3
Others^*c^	158	19.4
**Paternal main occupation**		
Farmer	376	46.3
Employee (government/private)	297	36.6
Others^*d^	139	17.1
**Wealth status (of the family)**		
Poor	270	33.2
Medium	267	32.9
Rich	275	33.9

*a *Catholic/Wakefata*.

*b *Tigre/Gurage*.

*c *Merchant/daily labor*.

*d *Merchant/daily laborer*.

Nearly one-third, 264 (32.5%) of the participants had sexual experience. Over three-fourth (77.6%) of participants visited health facilities in the university; 35.6% were counseled on reproductive health issues; 33.7% of participants used reproductive healthcare; and 49.1% of them discussed reproductive health issues with their parents (Table 2).

**Table 2:** Reproductive health related factors of Haramaya University students, Eastern Ethiopia, 2018 (*n* = 812).

**Characteristic**	**Frequency**	**Percent**
**History of sexual intercourse**		
Yes	264	32.5
No	548	67.5
**If yes, what was your age at first sexual intercourse (in years) (*****n*** **= 264)**		
<15	12	2.6
15–20	149	56.4
20–24	101	38.3
>24	2	0.8
**If yes, when did you started first sexual intercourse (*****n*** **= 264)**		
Before join university	170	64.4
After join university	94	35.6
**Number of sexual partners since first sex (*****n*** **= 264)**		
One	135	16.6
Two	57	7.0
Three	30	3.7
Four and above	42	5.2
**Advised about RH issues**		
Yes	318	39.2
No	494	60.8
**Utilization of RH services**		
Yes	274	33.7
No	538	66.3
**Participation in RH clubs**		
Yes	186	22.9
No	626	77.1
**Discussion about RH issues**		
Yes	650	80.0
No	162	20.0
**Heard information about RH issues**		
Yes	801	98.6
No	11	1.4
**Source of information about RH issues for first time (*****n*** **= 801)**		
Parents	136	17.0
Peer	299	37.3
Healthcare worker	86	10.7
School teacher	136	17.0
Medias (television, social media, and other media)	144	18.0

*RH, Reproductive Health*.

### Knowledge of Reproductive Rights

Initially, we checked for the internal consistency among 24 yes/no items used to assess the level of knowledge of reproductive rights. We found internally consistent items (Cronbach's α = 0.73). The mean (±SD) score of knowledge of reproductive rights was 14.36 ± 3.65. About 424 (52.2%) participants knew reproductive rights (95% CI: 48.8, 55.4%) in the setting.

### Predictors of Knowledge of Reproductive Rights

In the bivariable analysis, residence area, maternal education, type of the school, year of the study, faculty, and wealth status, utilization of reproductive health service and participating on the reproductive health club, counseling on reproductive health issues, and discussion on reproductive health issue were associated with knowledge of reproductive rights at *P*-value < 0.05. All independent variables with *P*-value < 0.25 in the bivariable analysis were included in our multivariable analysis model (Table 3).

**Table 3:** Factors associated with knowledge of reproductive rights among Haramaya University students, Eastern Ethiopia, 2018 (*n* = 812).

**Characteristic**	**Knowledge of reproductive rights**		**COR (95% CI)**	**AOR (95% CI)**
	**Yes, *n* (%)**	**No, *n* (%)**		
**Age (in years)**				
≤ 19	24 (42.1)	33 (57.9)	1	1
20–24	392 (52.8)	350 (47.2)	1.54 (0.89, 2.66)	1.38 (0.73, 2.62)
≥25	8 (61.5)	5 (38.5)	2.20 (0.64, 7.56)	1.24 (0.32, 4.84)
**Sex**				
Male	264 (50.7)	257 (49.3)	0.84 (0.63, 1.12)	0.67 (0.43, 1.36)
Female	160 (55.0)	131 (45.0)	1	1
**Residence area**				
Urban	219 (59.0)	152 (41.0)	1.66 (1.26, 2.19)***	1.53 (0.98, 1.87)
Rural	205 (46.5)	236 (53.5)	1	1
**Type of school attended before joining University**				
Private	65 (59.1)	45 (40.9)	1.50 (0.99, 2.27)*	1.48 (0.93, 2.35)
Both	69 (62.7)	41 (37.3)	1.75 (1.15, 2.66)**	1.01 (0.62, 1.63)
Government	290 (49.0)	302 (51.0)	1	1
**Faculty of the study**				
Health	100 (75.2)	33 (24.8)	3.22 (2.18, 5.06)***	3.37 (2.17, 5.23)***
Others^*a^	324 (47.7)	355 (52.3)	1	1
**Year of the study**				
Fourth and above	138 (64.5)	76 (35.5)	2.62 (1.79, 3.84)***	2.37 (1.58, 3.54)***
Third	90 (50.3)	89 (49.7)	1.46 (0.99, 2.16)	1.37 (0.91, 2.08)
Second	101 (54.0)	86 (46.0)	1.69 (1.15, 2.50)**	1.57 (1.05, 2.36)*
First	95 (40.9)	137 (59.1)	1	1
**Maternal educational level**				
Above secondary	77 (66.4)	39 (33.6)	2.36 (1.52, 3.66)***	1.42 (0.81, 2.55
Secondary	81 (61.8)	50 (38.2)	1.94 (1.28, 2.92)**	1.41 (0.87, 2.31)
Primary	107 (49.5)	109 (50.5)	1.17 (0.84, 1.65)	1.10 (0.74, 1.62)
No formal education	159 (45.6)	190 (54.4)	1	1
**Paternal educational level**				
Above secondary	147 (63.6)	84 (36.4)	1.89 (1.27, 2.80)**	1.89 (1.27, 2.80)**
Secondary	72 (48.3)	77 (51.7)	1.01 (0.66, 1.56)	1.01 (0.66, 1.56)
Primary	117 (47.0)	132 (53.0)	0.96 (0.65, 1.40)	0.96 (0.65, 1.40)
No formal education	88 (48.1)	95 (51.9)	1	1
**Wealth index (of the family)**				
Rich	156 (56.7)	119 (43.3)	1.54 (1.10, 2.16)*	1.54 (1.07, 2.21)*
Medium	144 (53.9)	123 (46.1)	1.38 (0.98, 1.94)	1.44 (1.02, 2.08)*
Poor	124 (45.9)	146 (54.1)	1	1
**Advised about RH issues**				
Yes	188 (59.1)	130 (40.9)	1.58 (1.19, 2.10)**	1.32 (0.93, 1.86)
No	236 (47.8)	258 (52.2)	1	1
**Utilization of RH services**				
Yes	195 (71.1)	79 (28.9)	2.76 (2.21, 4.98)***	2.81 (2.21, 4.98)***
No	236 (43.9)	302 (56.1)	1	1
**Participation in RH club**				
Yes	137 (62.6)	82 (37.4)	1.78 (1.30, 2.45)***	1.77 (1.27, 2.47)***
No	287 (48.4)	306 (51.6)	1	1
**Discuss about RH issues**				
Yes	355 (54.6)	295 (45.4)	1.62 (1.15, 2.30)**	1.31 (0.88, 1.94)
No	69 (42.6)	93 (57.4)	1	1

*Significant at*

* *P < 0.05, at*

** *P < 0.01*,

*** *P < 0.001*.

*RH, Reproductive Health*.

*a *All other faculty/colleges and Institute of Technology*.

In the multivariable analysis, the odds of knowledge of reproductive rights was two [AOR = 2.37 (1.58, 3.54)] and one and a half [AOR = 1.57 (1.05, 2.36)] times higher among students in the second and fourth and above year of the study compared to students in the first year. The odds of knowledge of reproductive rights was nearly two times [AOR = 1.89 (1.27, 2.80)] higher among students whose fathers had higher education compared to those whose fathers had no formal education. The odds of knowledge of reproductive rights was 44% [AOR = 1.44 (1.02, 2.08)] and 54% [AOR = 1.54 (1.07, 2.21)] higher among students who came from medium and rich families compared to those who came from poor families. The odds of knowledge of reproductive rights was three times [AOR = 3.37 (2.17, 5.23)] higher among students who were in the health faculty compared to those in non-health faculties. The odds of knowledge of reproductive rights was nearly three times [AOR = 2.81 (2.21, 4.98)] higher among students who ever used reproductive health services compared to those who did not use them. The odds of knowledge of reproductive rights was nearly two times [AOR = 1.77 (1.27, 2.47)] higher among students who participated in reproductive health clubs compared to those who did not (Table 3).

## Discussion

We investigated the magnitude of knowledge of reproductive rights and associated factors among Haramaya University students. In this study, more than half (52.2%) of the participants know about reproductive rights. Knowledge of reproductive rights among Haramaya University students was positively and significantly associated with being in the fourth and above year of the study, paternal higher education, being from the health faculty, coming from the rich and medium families, utilization of reproductive health service, and participation in the reproductive health club.

This study revealed that around one out of two (52.2%) students knew reproductive rights. This finding is somewhat similar to institution-based cross-sectional studies conducted in Wolaita Sodo, southern Ethiopia (54.5%) (13), southwest Nigeria (60.3%) (19), and Nepal (83.3%) (25). However, this finding is higher than a community-based cross-sectional study conducted in the Shire, northern Ethiopia (47.1%) (26). This variation might be explained by the methodological differences across the conducted studies. For instance, the Nigerian study used fewer items to measure the level of knowledge of reproductive rights (19), and also the Nepal study with better sexual reproduction knowledge use the scale with a lower number of items to assess the knowledge of reproductive rights (25). Moreover, the observed variation could also be due to the differences in the study settings and the differences in educational status, set-up, and sociocultural characteristics of the study population across the studies.

In this study, knowledge of reproductive rights was 2-folds higher among students in the fourth and above years of the study compared to those in the first year. This could be explained by the fact that the chance of getting information, and participating in and discussing reproductive health issues rise as the duration of stay in the campus increased as seen in the Shire, northern Ethiopia (26), and Thailand (27).

Paternal educational level has a significant association with knowledge of reproductive rights. Students whose fathers had higher education were nearly 2-folds more likely to know reproductive rights than those whose fathers had no education. This is supported by a cross-sectional study conducted in Riyadh, Saud Arabia revealed that students who discussed with educated families were more likely to know reproductive rights than students who discussed with uneducated families (16).

In this study, the wealth status of the family had a significant association with knowledge of reproductive rights. Knowledge of reproductive rights was around 2- and 3-folds higher when the participants came from medium and rich families compared to those who came from poor families. This finding was lower than the cross-sectional study conducted in East Gojjam, northern Ethiopia, which was 3-folds higher odds of knowledge of reproductive rights (15). This discrepancy in the effect size between studies could be since that students who had a family with a higher socioeconomic class could have a higher income and a better chance of access to different media and information. In addition, this finding could be supported by the cross-sectional study conducted in Wolaita Sodo, southern Ethiopia which reported students who came from the urban area were more likely to know reproductive rights than those who came from the rural area. Since most of the urban populations are high in economic status than rural populations (13).

In this study, knowledge of reproductive rights was 3-folds higher among students who were in the health faculty compared to the non-health faculties. This is consistent with the cross-sectional study done in Wolaita Sodo University, which reported 3-folds higher knowledge of reproductive rights among students who come from the health faculty (13).

In this study, uptake of reproductive health services was positively and significantly associated with the higher odds of knowledge of reproductive rights. Students who used reproductive health services were three times more likely to know reproductive rights. This was lower than the finding of a cross-sectional study done in southern Ethiopia and the possible reason for the discrepancy could be due to the higher chance of receiving counseling by healthcare workers before utilization and giving more attention to know what they use (13).

In this study, participating in the reproductive health club was associated with nearly 2-folds higher odds of knowledge of reproductive rights. This could be since those who participated in reproductive health clubs could get new and updated information and policies established on reproductive health and related rights early on time which supported by cross-sectional studies done in South Africa (4), the Shire, northern Ethiopia (26) and Wolaita Sodo, southern Ethiopia (13).

As a strength, this study used a large sample size and multistage stratified sampling given we included participants from different parts of the country. This study also shared the limitation of cross-sectional studies; the difficulty of determining the causal relationship between variables and the study findings also could not be generalized to undergraduate students in private universities in Ethiopia. The cross-sectional studies require the potential abilities of respondents to remember information retrospectively, recall bias also other limitations of this study. However, scientific procedures were employed to minimize possible effects. In addition, a pre-test of the data tool, supervision, and adequate training for data collectors and supervisors were utilized.

## Conclusions

This study revealed that about half (52.2%) of Haramaya University students knew reproductive rights. In this study, being in the fourth and above year of the study, paternal higher education, being from medium and rich families, in the health faculty, using reproductive health services, and participating in reproductive health clubs were factors associated with knowledge of reproductive rights. Based on the findings, Ethiopian higher education institutions should collaborate with the ministry of health in providing reproductive and sexual rights-related courses for non-health discipline students. The district health office should collaborate with Haramaya University in providing quality reproductive health services for youth students. Public health facilities at different levels should encourage students to actively participate in reproductive health issues in collaboration with existing higher education facilities. Healthcare service providers at all levels should focus on providing quality reproductive health services and arranging special education and counseling programs for all discipline students on sexual and reproductive rights. Very importantly, continuous support and promotion of youth health clubs would be essential, sustainable, and feasible options to promote knowledge and access to sexual and reproductive healthcare services and rights.

## Data Availability Statement

The raw data supporting the conclusions of this article will be made available by the authors, without undue reservation.

## Ethics Statement

The studies involving human participants were reviewed and approved by Institutional Health Research Ethical Review Committee of College of Health and Medical Sciences, Haramaya University approved the protocol of the study (Ref. No: IHRERC/028/2018). The patients/participants provided their written informed consent to participate in this study.

## Author Contributions

MY, HAA, NA, LO, AA, and AAU participated in the conception of the idea, designing the study, data collection and analysis, writing up the draft results, reanalyzed the data, drafted, edited, and revised the manuscript. All authors agree to take responsibility and be accountable for the contents of the article, agree on the journal to which the article will be submitted, and read and approved the final manuscript.

## Conflict of Interest

The authors declare that the research was conducted in the absence of any commercial or financial relationships that could be construed as a potential conflict of interest.

## Publisher's Note

All claims expressed in this article are solely those of the authors and do not necessarily represent those of their affiliated organizations, or those of the publisher, the editors and the reviewers. Any product that may be evaluated in this article, or claim that may be made by its manufacturer, is not guaranteed or endorsed by the publisher.

## Abbreviations

AOR: adjusted odds ratio
HIV: human immune deficiency virus
IHRERC: Institution Health Research Ethical Review Bureau
RH: reproductive health
WHO: World Health Organization.
