# Supplementary material for: Level and Predictors of Knowledge of Reproductive Rights Among Haramaya University Students, Eastern Ethiopia: A Cross-Sectional Study
Source: Front Reprod Health. 2021 Nov 24;3:641008. doi: 10.3389/frph.2021.641008 (PMC9580644; doi:10.3389/frph.2021.641008)
Supplement: Supplementary file 1 [file Data_Sheet_1.pdf]

Supplementary File 1: Sampling scheme for the study on knowledge of reproductive rights among Haramaya University students, eastern Ethiopia, 2018

Colleges	Year I	Year II	Year III	Year IV+	Total
	Population (Sample)	Population (Sample)	Population (Sample)	Population (Sample)	Population (Sample)
Natural and computational science	268 (13)	335 (17)	249 (12)	0 (0)	852 (42)
Agriculture and environmental science	637 (32)	580 (29)	470 (24)	0 (0)	1687 (84)
Veterinary medicine	51 (2)	43 (2)	37 (2)	91 (5)	222 (11)
Business and economics	542 (27)	400 (20)	326 (16)	0 (0)	1268 (64)
Computing and Informatics	447 (22)	338 (17)	320 (16)	237 (12)	1342 (67)
Education and behavioral sciences	267 (14)	206 (10)	137 (7)	0 (0)	610 (31)
Social science and humanities	517 (26)	426 (21)	287 (14)	19 (1)	1249 (62)
Law	107 (5)	66(4)	79(4)	143 (7)	395 (20)
Health and Medical sciences	527 (27)	600 (30)	509 (26)	1018 (51)	2654 (133)
Institute of Technology	1384 (69)	869 (43)	1058 (53)	2847 (142)	6158 (308)
Subtotal	4747 (237)	3863 (193)	3472(174)	4355 (218)	16437 (822)
