# Supplementary material for: Level and Predictors of Knowledge of Reproductive Rights Among Haramaya University Students, Eastern Ethiopia: A Cross-Sectional Study
Source: Front Reprod Health. 2021 Nov 24;3:641008. doi: 10.3389/frph.2021.641008 (PMC9580644; doi:10.3389/frph.2021.641008)
Supplement: Supplementary file 2 [file Data_Sheet_2.doc]

## Supplementary File 2: English version Questionnaire

| 1. **General Information** | | | |
| --- | --- | --- | --- |
| ***s.n*** | **Characteristics** | **Responses** | **Skip to** |
| **100** | Identification number | **____________________________** |  |
| **101** | Date of interview (dd/mm/yyyy) | **____________________________** |  |
| 1. **Socio demographic and related information** | | | |
| 200 | How old are you? (Age in complete years) | ____________________________ |  |
| 201 | What is your sex? | 1. Female 2. Male |  |
| 202 | What is your religion? | 1. Orthodox 2. protestant 3. Muslim 4. Other/specify |  |
| 203 | What is your current marital status? | 1. Single 2. married 3. divorced 4. separated 5. widowed |  |
| 204 | What is total number of persons living in your family | _________ |  |
| 205 | Where did you come from? | 1. Urban 2. Rural |  |
| 206 | What is your ethnic origin? |  |  |
| 207 | Type of school you have attended before joining university | 1. Governmental 2. Private 3. Both |  |
| 208 | What is your faculty? |  |  |
| 209 | What is your year of study? | 1. First year 2. Second year 3. Third year 4. Fourth year and above |  |
| 210 | what is the highest educational level of your father | 1. No formal education 2. Elementary school 3. Secondary school 4. College and above |  |
| 211 | What is your father’s work status? | 1. Governmental employee 2. Private employee 3. Trader 4. Farmer 5. Others/specify |  |
| 212 | What is the highest educational level of your mother? | 1. No education 2. Elementary school 3. Secondary school 4. college and above |  |
| 213 | What is your mother’s main occupation/work? | 1. Housewife 2. Governmental employee 3. private employee 4. farmer 5. Others, specify________ |  |

| **Questions to assess the current family wealth/economic condition:**  **(**Could you tell me if you have the following in your house?) | | | |
| --- | --- | --- | --- |
|  | **Asset type** | **Response** | |
|  | **Domestic animals** |  |  |
| 214 | Ox | No (0) | Yes (1) |
| 215 | Cow | No (0) | Yes (1) |
| 216 | Calf | No (0) | Yes (1) |
| 217 | Sheep | No (0) | Yes (1) |
| 218 | Goat | No (0) | Yes (1) |
| 219 | Horse | No (0) | Yes (1) |
| 230 | Donkey | No (0) | Yes (1) |
| 231 | Cock and Hen | No (0) | Yes (1) |
|  | **Durable assets** |  |  |
| **232** | Television | No (0) | Yes (1) |
| 233 | Radio | No (0) | Yes (1) |
| 234 | Electricity | No (0) | Yes (1) |
| 235 | Refrigerator | No (0) | Yes (1) |
| 236 | Conventional telephone | No (0) | Yes (1) |
| 237 | Mobile phone | No (0) | Yes (1) |
| 238 | Car | No (0) | Yes (1) |
| 239 | Motorcycle | No (0) | Yes (1) |
| 240 | Cycle | No (0) | Yes (1) |
| 241 | Cart | No (0) | Yes (1) |
| 242 | Gold, money | No (0) | Yes (1) |
| 243 | Ownership of the owned living house | No (0) | Yes (1) |
| 244 | Ownership of agricultural land | No (0) | Yes (1) |
|  | **Productive assets** |  |  |
| 245 | Plough plow | No (0) | Yes (1) |
| 246 | Axe | No (0) | Yes (1) |
| 247 | Hoe | No (0) | Yes (1) |
| 248 | Shovel | No (0) | Yes (1) |
| 249 | Sickle | No (0) | Yes (1) |
| 250 | Modern beehive | No (0) | Yes (1) |
| 251 | Traditional beehive | No (0) | Yes (1) |
|  | **Housing characteristics** |  |  |
| 252 | Indoor plumping/ pipe water | No (0) | Yes (1) |
| 253 | Type of flooring | Earth/dung (0) | Cement/raw wood (1) |
| 254 | Toilet facility | Unsanitary or traditional pit latrine/ no toilet (0) | Sanitary or improved pit latrine (1) |
|  | **Other household materials** |  |  |
| 255 | Sofa | No (0) | Yes (1) |
| 256 | Bed | No (0) | Yes (1) |
| 257 | Table | No (0) | Yes (1) |
| 258 | Chair | No (0) | Yes (1) |
| 259 | Stove | No (0) | Yes (1) |

| 1. **Sexual and reproductive factors** | | | |
| --- | --- | --- | --- |
| 300 | Have you ever had sexual intercourse? | 1. Yes 2. No | If no, skip to 304 |
| 301 | If yes, at what age did you have the first sexual intercourse (Age in complete years) | ______ |  |
| 302 | If yes, when did you start sexual intercourse? | 1. Before joining university 2. After joined the university |  |
| 303 | If yes, how many sexual partners have you ever had in your lifetime? | _____________________ |  |
| 304 | Do you ever participate in reproductive health clubs? | 1. Yes 2. No |  |
| 305 | Do you ever advised/counselled about reproductive health issues? | 1. Yes 2. No |  |
| 306 | Do you ever utilized sexual and reproductive health services? | 1. Yes 2. No |  |
| 307 | Is it important to discuss/talk about reproductive issues with somebody else? | 1. Yes 2. No |  |
| 308 | Have you ever discussed about reproductive issues with anyone else? | 1. Yes 2. No | If no, skip to 310 |
| 309 | If yes, with who had you discussed reproductive issues for the first time? | 1. With my Mother 2. With my father 3. With my sister 4. With my friends 5. With my school teacher 6. With Health personnel 7. With other, specify___ |  |
| 310 | Have you ever heard information about reproductive health issues? | 1. Yes 2. No | If no, skip to 400 |
| 311 | What is your source of information on reproductive and sexual health? | 1. Parents 2. Peer 3. School teacher 4. Internet 5. Health personnel 6. Media RH clubs 7. University student clinic 8. Others specify____ |  |

| 1. **QUESTIONS ABOUT KNOWLEDGE OF REPRODUCTIVE RIGHTS** | | | |
| --- | --- | --- | --- |
| 400 | Do families have right to decide about their female child to be circumcised? | 1.Yes 2. No |  |
| 401 | Can a girl dismiss her arranged marriage without her families’ agreement? | 1.Yes 2. No |  |
| 402 | Do youths have right to mate selection without their families consent? | 1.Yes 2. No |  |
| 403 | Can a married woman say no to have children if she doesn’t want? | 1.Yes 2. No |  |
| 404 | Does a woman have a right to say no to have sex, regardless of her boyfriend’s wishes? | 1.Yes 2. No |  |
| 405 | Do youths have the right that their use of reproductive health services is to be kept confidential? | 1.Yes 2. No |  |
| 406 | Should a man get sex whenever he wants irrespective of his girlfriend’s wish? | 1.Yes 2. No |  |
| 407 | Do youths have the right to ask each other for HIV testing before sexual engagements? | 1.Yes 2. No |  |
| 408 | Do you think youths have the right to information on reproductive health facilities? | 1.Yes 2. No |  |
| 409 | Do you think that youths have the right to be free from all forms of discrimination because of their reproductive and sexual orientation? | 1.Yes 2. No |  |
| 410 | Do boys have no special right to be protected from sexual exploitation and abuse? | 1.Yes 2. No |  |
| 411 | Do youths have rights to get knowledge of reproductive rights at higher institutions? | 1.Yes 2. No |  |
| 412 | Do all girls have the right to access to autonomous reproductive choices including choices relating to safe abortion? | 1.Yes 2. No |  |
| 413 | Do girls have the right to resist genital mutilation against their families will? | 1.Yes 2. No |  |
| 414 | Do youths have a full right to access all reproductive health services without parents’ consent? | 1.Yes 2. No |  |
| 415 | Do youths have the right to decide on sexual and reproductive health issues of themselves without their parents’ consent? | 1.Yes 2. No |  |
| 416 | Do all women have the right to autonomous reproductive choices to use any type of contraceptives? | 1.Yes 2. No |  |
| 417 | Do girls have the right to autonomous reproductive choices without their partner's consent? | 1.Yes 2. No |  |
| 418 | Do students have a right to freedom of assembly and political participation to influence governments to place priority on sexual & reproductive health? | 1.Yes 2. No |  |
| 418 | Do students have the right to access new reproductive technologies? | 1. Yes 2. No |  |
| 420 | Do you think that all students must be free to enjoy and control their sexual and reproductive life? | 1.Yes 2. No |  |
| 421 | Do youths have the right to form an association or clubs that aims to promote their sexual and reproductive health? | 1.Yes 2. No |  |
| 422 | Do unmarried woman have the right to maternity leave with adequate social security benefits? | 1.Yes 2. No |  |
| 423 | Do unmarried couples have right to use contraceptives without condoms? | 1. Yes 2. No |  |
